# Early Patterns of Macular Degeneration in *ABCA4*-Associated Retinopathy

**DOI:** 10.1016/j.ophtha.2017.11.020

**Published:** 2018-05

**Authors:** Kamron N. Khan, Melissa Kasilian, Omar A.R. Mahroo, Preena Tanna, Angelos Kalitzeos, Anthony G. Robson, Kazushige Tsunoda, Takeshi Iwata, Anthony T. Moore, Kaoru Fujinami, Michel Michaelides

**Affiliations:** 1 Department of Genetics, University College London Institute of Ophthalmology, University College London, London, United Kingdom; 2 Medical Retina Service, Moorfields Eye Hospital, London, United Kingdom; 3 Department of Ophthalmology, Leeds Institute of Molecular Medicine, St James's University Hospital, Leeds, United Kingdom; 4 Department of Electrophysiology, Moorfields Eye Hospital, London, United Kingdom; 5 Division of Vision Research, National Institute of Sensory Organs, National Hospital Organization, Tokyo Medical Center, Tokyo, Japan; 6 Division of Cellular and Molecular Biology, National Institute of Sensory Organs, National Hospital Organization, Tokyo Medical Center, Tokyo, Japan; 7 Department of Ophthalmology, University of California San Francisco Medical School, San Francisco, California; 8 Department of Ophthalmology, Keio University School of Medicine, Tokyo, Japan

## Abstract

**Purpose:**

To describe the earliest features of *ABCA4*-associated retinopathy.

**Design:**

Case series.

**Participants:**

Children with a clinical and molecular diagnosis of *ABCA4*-associated retinopathy without evidence of macular atrophy.

**Methods:**

The retinal phenotype was characterized by color fundus photography, OCT, fundus autofluorescence (FAF) imaging, electroretinography, and in 2 patients, adaptive optics scanning laser ophthalmoscopy (AOSLO). Sequencing of the *ABCA4* gene was performed in all patients.

**Main Outcome Measures:**

Visual acuity, OCT, FAF, electroretinography, and AOSLO results.

**Results:**

Eight children with *ABCA4*-associated retinopathy without macular atrophy were identified. Biallelic variants in *ABCA4* were identified in all patients. Four children were asymptomatic, and 4 reported loss of VA. Patients were young (median age, 8.5 years; interquartile range, 6.8 years) with good visual acuity (median, 0.155 logarithm of the minimum angle of resolution [logMAR]; interquartile range, 0.29 logMAR). At presentation, the macula appeared normal (n = 3), had a subtly altered foveal reflex (n = 4), or demonstrated manifest fine yellow dots (n = 1). Fundus autofluorescence identified hyperautofluorescent dots in the central macula in 3 patients, 2 of whom showed a normal fundus appearance. Only 1 child had widespread hyperautofluorescent retinal flecks at presentation. OCT imaging identified hyperreflectivity at the base of the outer nuclear layer in all 8 patients. Where loss of outer nuclear volume was evident, this appeared to occur preferentially at a perifoveal locus. Longitudinal split-detector AOSLO imaging in 2 individuals confirmed that the greatest change in cone spacing occurred in the perifoveal, and not foveolar, photoreceptors. Electroretinography showed a reduced B-wave–to–A-wave ratio in 3 of 5 patients tested; in 2 children, recordings clearly showed electronegative results.

**Conclusions:**

In childhood-onset *ABCA4*-associated retinopathy, the earliest stages of macular atrophy involve the parafovea and spare the foveola. In some cases, these changes are predated by tiny, foveal, yellow, hyperautofluorescent dots. Hyperreflectivity at the base of the outer nuclear layer, previously described as thickening of the external limiting membrane, is likely to represent a structural change at the level of the foveal cone nuclei. Electroretinography suggests that the initial site of retinal dysfunction may occur after phototransduction.

Stargardt disease (STGD1; Online Mendelian Inheritance in Man identifier, 248200) is an autosomal recessive retinal dystrophy resulting from dysfunction in the photoreceptor-specific ATP-binding cassette transporter *ABCA4*. To date, more than 1000 mutations in *ABCA4* have been reported, associated with phenotypes that include macular, cone, or cone–rod dystrophies.^1^ Symptoms may develop as early as the first decade of life, but more commonly occur in the second or third decade. A minority of individuals demonstrate a late-onset form of disease in their forties or fifties that typically spares the central fovea.^2^ For the youngest patients, the earliest signs of disease are poorly described, and despite some understanding of the protein function, the precise pathogenic mechanisms are yet to be elucidated. As therapeutic clinical trials either already have commenced (e.g., stem cell transplantation [clinicaltrials.gov identifier, NCT01345006], modified vitamin A preparations [clinicaltrials.gov identifier, NCT02402660], and gene therapy [clinicaltrials.gov identifier, NCT01367444]) or are planned, there is a need to identify the site of retinal dysfunction in early disease and the pattern of progression. Because most reported cases of STGD1 manifest signs of macular atrophy at presentation, there is little information about earlier stages of disease. Herein, we present a series of patients with molecularly confirmed *ABCA4*-associated retinopathy without macular atrophy in which detailed retinal imaging and functional testing have provided new insights into the earliest stages of disease.

## Methods

Individuals with features suggestive of early *ABCA4*-associated retinopathy were recruited prospectively from the pediatric retinal genetics clinics at Moorfields Eye Hospital (MEH), London, United Kingdom, and Tokyo Medical Center, Tokyo, Japan. In addition, a retrospective case note review was performed at MEH to identify further participants. Those with macular atrophy at presentation were excluded from this study. Each patient underwent a full clinical examination, including Snellen or logarithm of the minimum angle of resolution (logMAR) visual acuity, color vision testing (Hardy Rand Rittler pseudoisochromatic color plate test, fourth edition), and dilated fundus examination. Retinal imaging included 35° color fundus photography (TRC-50DX; Topcon Corp, Tokyo, Japan) and 30° or 55° fundus autofluorescence (FAF)^3^ imaging (HRA2; Heidelberg Engineering, Ltd, Heidelberg, Germany). Spectral-domain OCT imaging was acquired using a Heidelberg HRA2 or Cirrus HD-OCT 500 (Cirrus; Carl Zeiss Meditec, Ltd, Dublin, CA) system. Adaptive optics scanning laser ophthalmoscopy (AOSLO) was carried out on 2 individuals using a custom-built instrument as previously described.^4^ Confocal and split-detector (nonconfocal) image sequences were acquired simultaneously.^4^, ^5^, ^6^ Parafoveal locations were imaged using either a 1° or 1.5° field of view up to 5° eccentricity from the fovea. The images were processed and a montage was created to illustrate a continuous photoreceptor mosaic. Retinal function was tested by full-field electroretinography and pattern electroretinography (PERG), obtained using either gold-foil electrodes, incorporating the current International Society for Clinical Electrophysiology of Vision (ISCEV) standards in 3 patients, or with Ganzfeld stimulation and lower eyelid skin electrodes in the 2 youngest children, according to pediatric protocols previously described.^7^, ^8^, ^9^ Genetic testing was performed by targeted exome sequencing for individuals at MEH (Stargardt/Macular dystrophy panel, version 3; Casey Eye Institute Molecular Diagnostics Laboratory, Portland, Oregon), or by whole exome sequencing for patients at Tokyo Medical Center as reported previously.^10^ Pathogenic variants were confirmed by Sanger sequencing. This study received institutional review board approval at Moorfields Eye Hospital, London, and adhered to the tenets of the Declaration of Helsinki. Patients or their legal guardians consented to the use of their clinical data for research purposes.

## Results

Eight individuals were identified who fulfilled the study criteria. Six were recruited prospectively and 1 was recruited retrospectively from MEH. Some clinical information for patient 7 has been published previously.^11^ The eighth patient was identified from the Inherited Eye Disease Service, National Hospital Organization, Tokyo Medical Center, Tokyo, Japan. The clinical and molecular genetic characteristics for all participants are presented in Table S1 (available at www.aaojournal.org).

Individuals were young (median age, 8.5 years; interquartile range, 6.8 years; range, 5–13 years) with good visual acuity (median, 0.15 logMAR; interquartile range, 0.29 logMAR; range, −0.14 to 0.3 logMAR) at their initial visit. Four patients showed symptoms at presentation, reporting a subjective decline in the quality of their vision. Four children with the best acuity were asymptomatic. These individuals were identified because of the presence of peripheral flecks (patient 3) or as a result of having a more severely affected elder sibling (patients 4, 6, and 8). Color vision was normal in all individuals tested. In most patients (6/8), the central macula either appeared normal or showed subtle alteration in the foveal reflex. One patient demonstrated yellow dots at the fovea (patient 6), whereas a further 2 children demonstrated these during follow-up (patients 7 and 8). In 7 of 8 patients, the peripheral retina appeared normal on slit-lamp biomicroscopy. Patient 3 showed widespread pisciform outer retinal flecks at presentation that spared the macula.

### Autofluorescence Imaging

**Figure 1:**
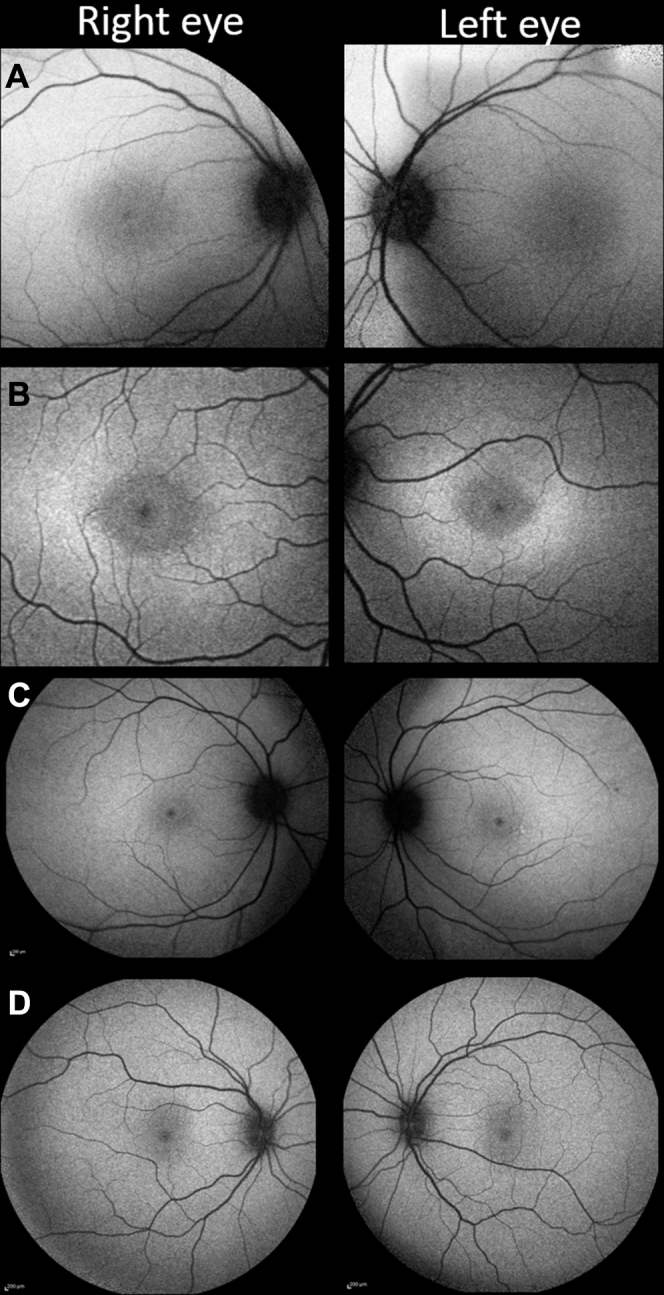
Fundus autofluorescence (FAF) changes in childhood-onset *ABCA4*-associated retinopathy. **A**, Patient 2 has a subtle increase in foveal FAF, while maintaining a small central zone of physiological hypoautofluorescence. **B**, In addition to the changes present in (**A**), a rhomboid zone of hyperautofluorescence is visible in patient 4. **C**, Peripheral macula imaging from patient 1 revealing a more extensive, diffuse hyperautofluorescent signal. **D**, A normal pattern of childhood autofluorescence shown for comparison.

**Figure 2:**
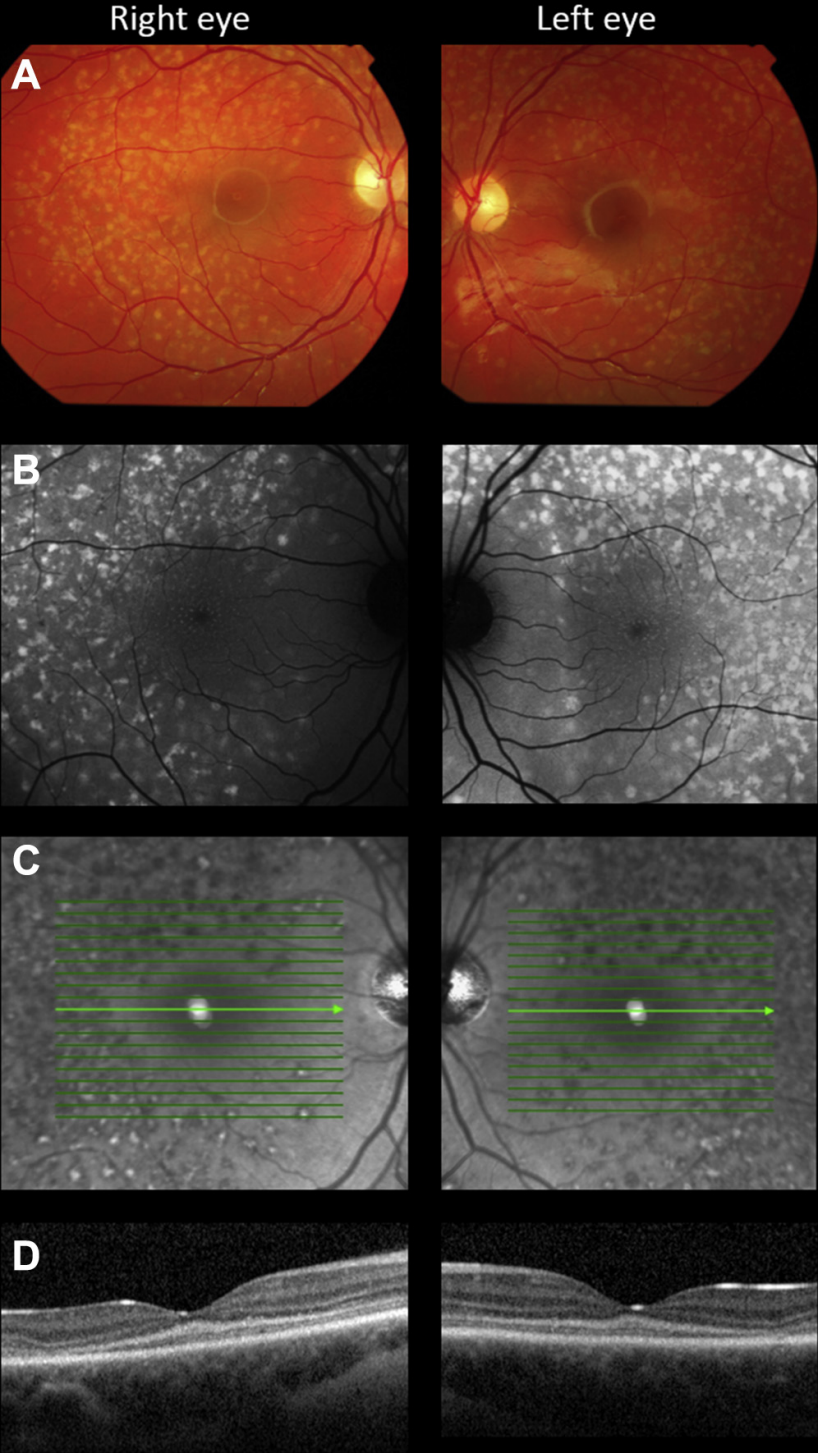
Multimodal imaging in patient 3. **A**, Color fundus photograph identifying multiple yellow flecks distributed throughout the posterior pole of both eyes, sparing the peripapillary retina. **B**, A 488-nm autofluorescence image revealing that the flecks are highly autofluorescent. In addition, numerous discrete autofluorescent dots are visible at the fovea, a feature of disease that usually is not observed. **C**, The flecks are predominantly hyporeflective, viewed using near infra-red light, but some appear to have a highly reflective center. **D**, OCT image (horizontal line scan through the foveola) demonstrating hyperreflectivity at the base of the outer nuclear layer and mild outer retinal degeneration in the temporal macula.

Fundus autofluorescence abnormalities were evident in all patients, even when funduscopy results appeared normal. All patients retained a variable degree of physiologic hypoautofluorescence at the foveola that qualitatively appeared to be reduced in size when compared with normal parameters (Fig 1A). The perifoveal retina was mildly hyperautofluorescent (Fig 1B). With increasing retinal eccentricity, a further band of hyperautofluorescence was evident in patients 1, 4, and 5, but less convincingly in patient 2 (Fig 1C). This was broader and more diffuse than the well-defined ring often observed in patients with other retinal dystrophies. At their first visit, numerous discrete hyperautofluorescent dots were present at the fovea in patients 3, 6, and 7 (Fig 2C). Patient 3 was the only patient to manifest hyperautofluorescent peripheral retinal flecks at presentation (Fig 2A). No patient demonstrated a complete loss of foveal autofluorescence, suggesting that the retinal pigment epithelium (RPE) remained intact at this early stage.

### OCT

**Figure 3:**
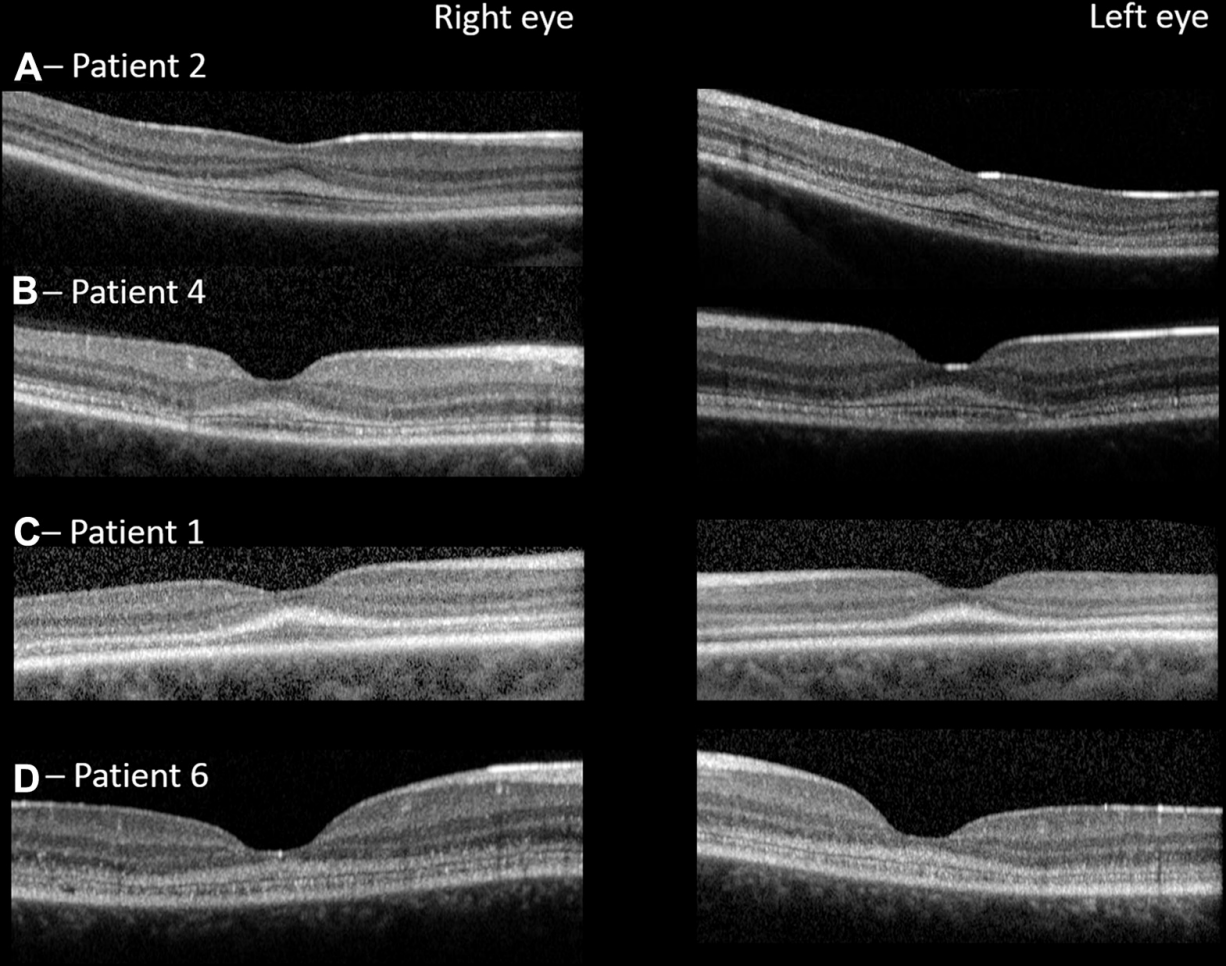
OCT images from 4 patients. Representative horizontal line scans through the foveola are shown for all patients. In all cases, there is a well-defined region of hyperreflectivity extending superiorly from the first highly reflective OCT line, the optical correlate of the external limiting membrane. It is maximally thick at the foveola, and reduces exponentially with increasing retinal eccentricity. In **A**–**D** the ellipsoid zone (second highly reflective outer retinal line) also is readily visible.

**Figure 4:**
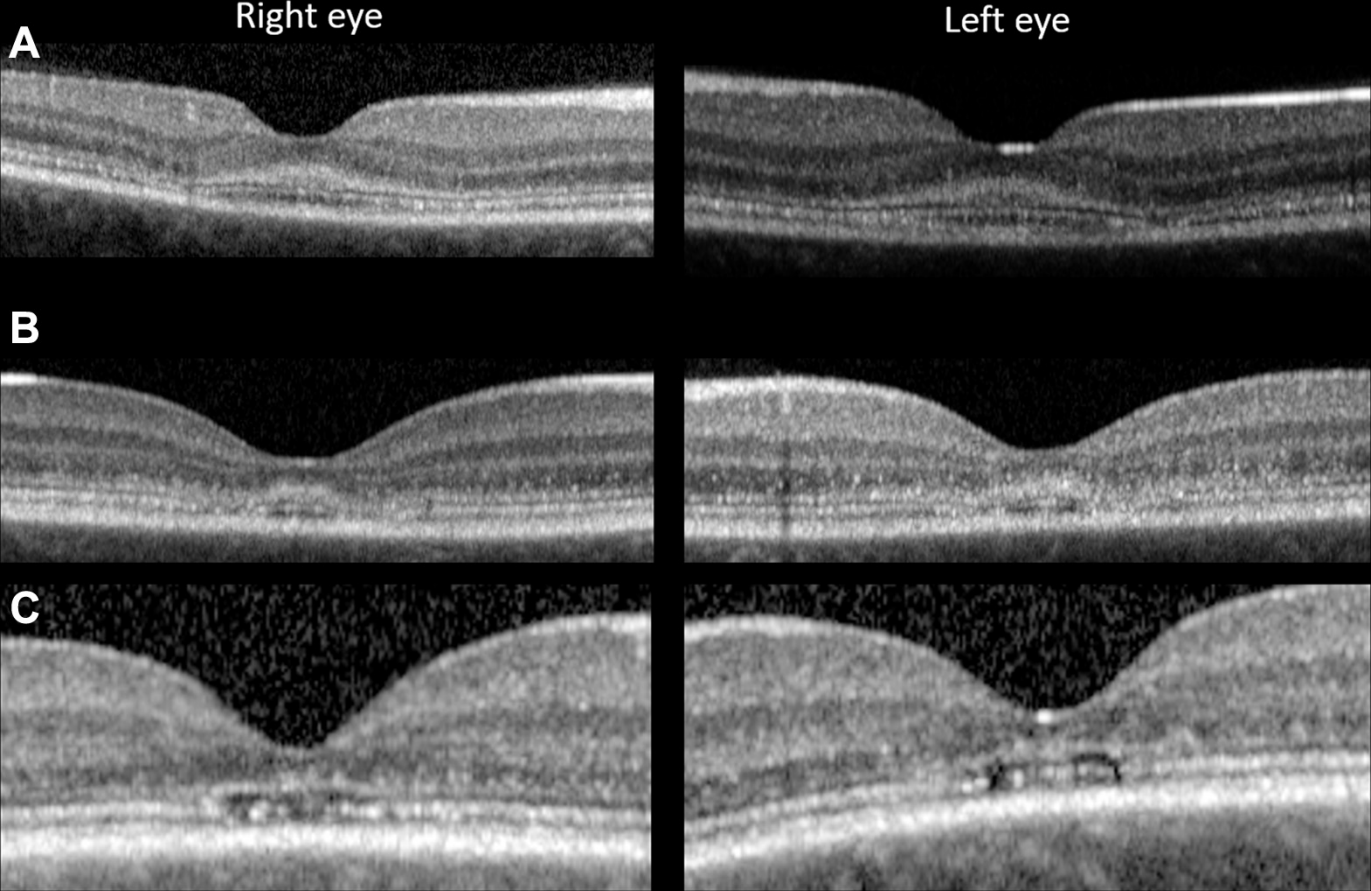
OCT images highlighting patterns of early foveal degeneration. Horizontal line scans through the foveola. **A**, Patient 4 with early temporal collapse bilaterally. The integrity of ellipsoid zone has been breached just temporal to the foveola. **B**, **C**, Imaging in patient 5 demonstrating the same changes nasally and temporally (**B**) at baseline and (**C**) 19 months later.

**Figure 5:**
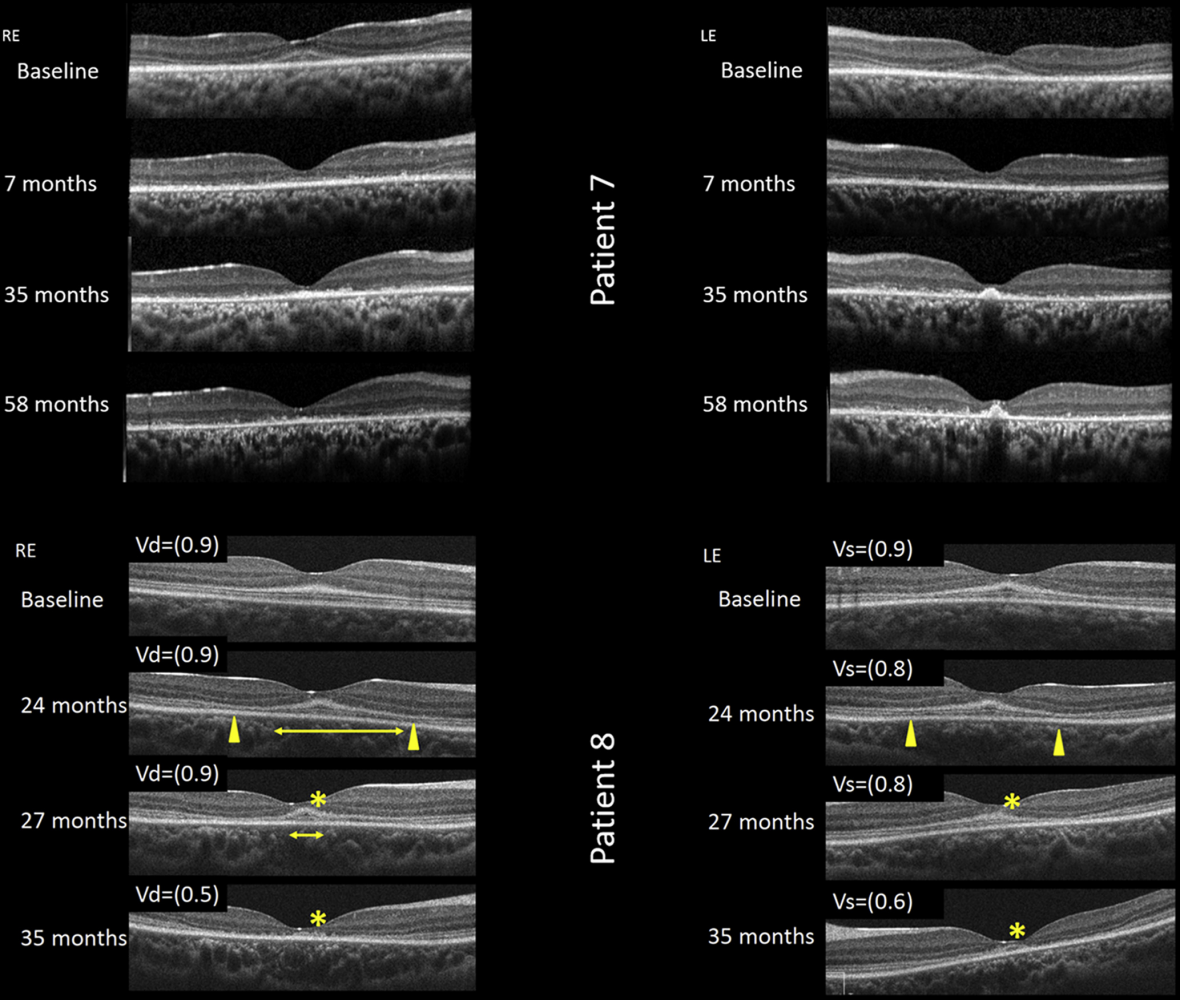
The evolution of macular atrophy in childhood-onset *ABCA4*-associated retinopathy. Sequential OCT (horizontal line scans through the foveola) in (upper) patient 7 and (lower, with decimalized visual acuity) patient 8. The earliest structural change appears to be hyperreflectivity at the base of the outer nuclear layer (patient 8, baseline), associated with near normal vision. Over the next 2 years, parafoveal outer retinal atrophy developed (*yellow arrowheads*), whereas the foveola is relatively spared of these changes (yellow horizontal line baseline images from patients 8 and 7). By month 35, the ellipsoid zone at the foveola clearly is degenerating, associated with a drop in acuity (*asterisk*, patient 8). LE = left eye; RE = right eye; Vd = visual acuity dexter; Vs = visual acuity sinister.

OCT imaging confirmed that the fourth highly reflective outer retinal band, thought to represent the RPE–Bruch's membrane^12^ complex, was well preserved in all individuals (Fig 3). Moreover, no areas of increased signal penetrance into the choroid were observed, in keeping with the absence of RPE atrophy or disease. The most prevalent finding, observed in all 8 patients, was a marked increase in reflectivity observed in the outer retina, commencing at the first highly reflective outer retinal band, thought to represent the external limiting membrane (ELM), and extending part way through the outer nuclear layer (ONL; Fig 3). This characteristic pattern of hyperreflectivity was highly reproducible and was evident in all patients, with a maximum thickness at the foveola, reducing nasally and temporally symmetrically, extending into the peripheral fovea. In most cases, the second highly reflective line thought to represent the ellipsoid zone was visible below this abnormally reflective band, but with reduced clarity, suggesting possible subtle disturbance to the photoreceptor outer segments (Fig 3). In all patients, focal collapse of the inner retinal layers was visible because of loss of outer retinal structures (Fig 4). This appeared to occur at perifoveal loci, rather than at the foveola. The first sign of outer retinal atrophy seen on OCT imaging appeared to spare the fovea. In keeping with this, sequential imaging over a 35- and 58-month period in 2 individuals (patients 8 and 7, respectively), demonstrated that the foveolar photoreceptors degenerated last (Fig 5). For patient 5, progressive loss of foveal outer segments occurred over a 19-month period, resulting in a broad outer retinal cavity (Fig 4B and C). Importantly, an intact ELM of physiologic proportions also was visible. Where the evolution of outer retinal degeneration was captured on OCT, the temporal perifovea always seemed to be affected first.

### Adaptive Optics Scanning Laser Ophthalmoscopy

**Figure 6:**
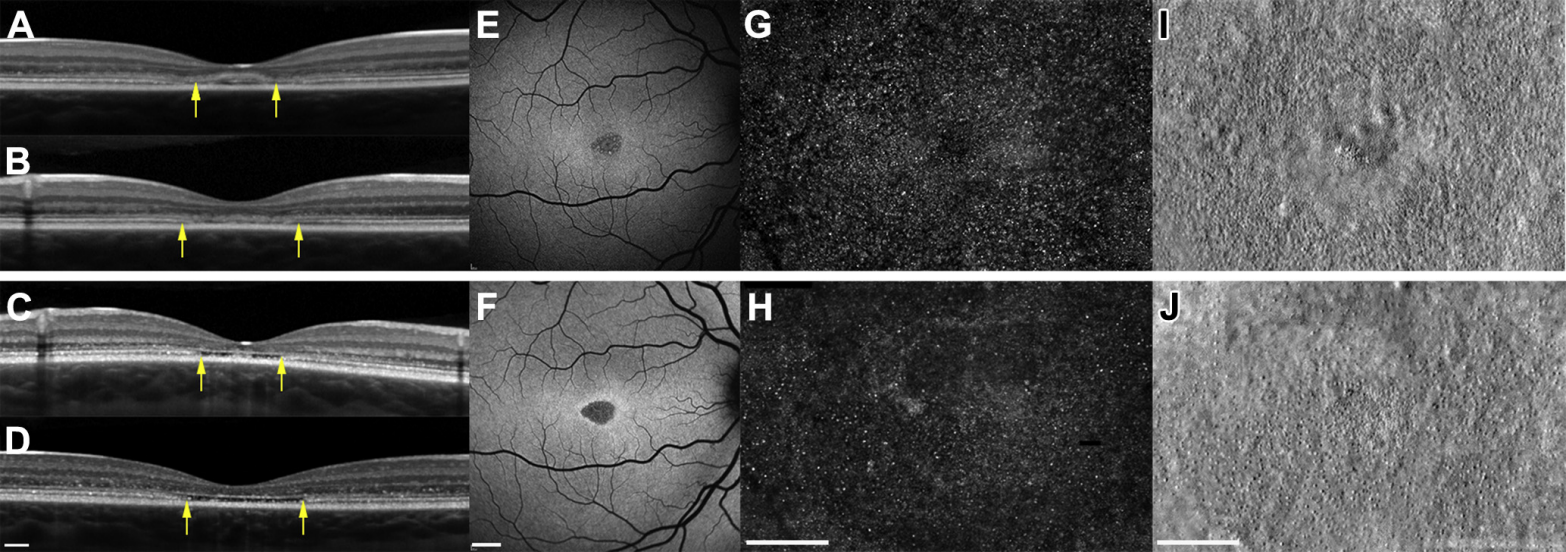
Spectrum of foveal structure shown with (**A**–**D**) OCT (scale bar, 200 μm), (**E**, **F**) fundus autofluorescence (scale bar, 1000 μm), and (**G**–**J**) adaptive optics scanning laser ophthalmoscopy (AOSLO; scale bar, 200 μm) at baseline (upper panel) and 19 months later (lower panel) for patient 5. *Arrows* denote outer edges of the region imaged on AOSLO. Vertical scan (**A**) and horizontal scan (**B**) demonstrating initial hyperreflectivity at the base of the outer nuclear layer and parafoveal outer retinal degeneration. Vertical scan (**C**) and horizontal scan (**D**) detail the evolution of outer retinal atrophy and parafoveal cavitation. **E**, **F**, Fundus autofluorescence over the same period. Adaptive optics scanning laser ophthalmoscopy imaging of the foveal cones at baseline (**G**, confocal; **I**, split-detector) and 19 months later (**H**, confocal; **J**, split-detector). **G**, **H**, Confocal imaging revealing areas of nonwaveguiding cones evident from both time points, predominantly in a parafoveal location, suggesting cell loss from baseline visit. **H**, However this is more apparent at the 19-month follow-up visit. Split-detector images show the diameter increase of inner segments and the increase in cell spacing over this period, again in a parafoveal location.

**Figure 7:**
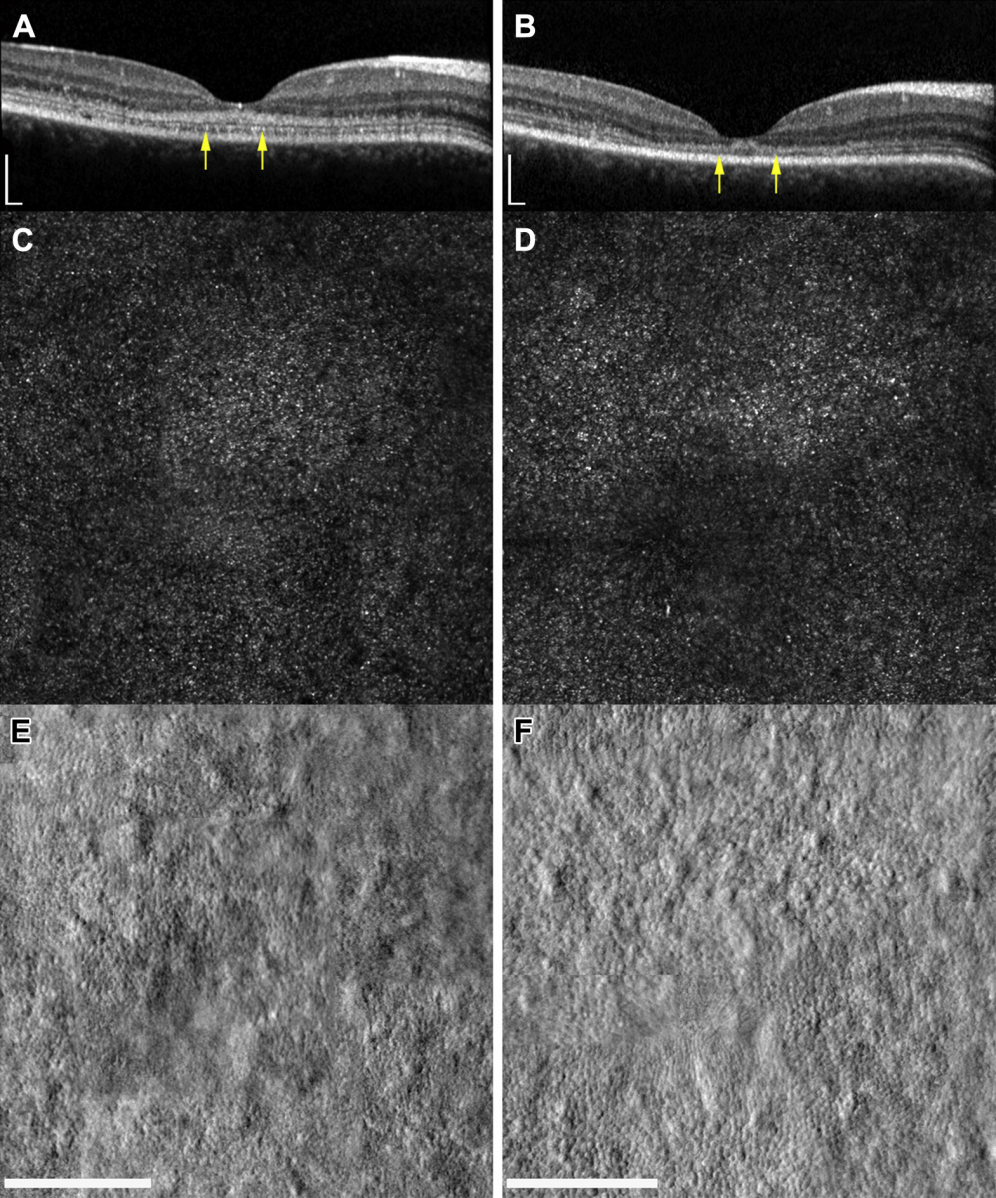
Foveal structure shown with (**A**, **B**) OCT and (**C**, **D**) adaptive optics scanning laser ophthalmoscopy at baseline (left hand column) and 13 months later (right hand column) for patient 6. Scale bars, 200 μm. *Arrows* denote outer edges of the region imaged on adaptive optics scanning laser ophthalmoscopy (AOSLO). Both (**C**, **D**) confocal and (**E**, **F**) split-detector imaging are presented. Confocal imaging reveals only subtle changes over this period, manifested by an increase in dark areas of nonwaveguiding cones. Split-detector imaging shows a more evident change in parafoveal cone structure over 13 months, with a marked increase in the diameter of cone inner segments.

Patients 5 and 6 underwent further retinal imaging of the photoreceptor layer of their right eyes using a custom-build AOSLO. Nineteen-month and 13-month follow-up data are shown in Figures 6 and 7, respectively. Structural changes to the cone photoreceptor cells of both children were identified. For patient 5, an annulus of nonuniform reflectance was visible, suggesting the first signs of possible outer segment loss in this area. Confocal imaging also revealed areas of nonwaveguiding cones in a 2° annulus around the foveola, with relative sparing of the central foveal cones, which appeared more contiguous and uniformly reflective than their parafoveal neighbors. The split detector-derived images also revealed abnormal, enlarged cone inner segments. The foveal center was relatively spared of these changes, because the cone inner segments remain tightly packed. This region of disturbance to the cone outer segments correlates well with the area of hyperautofluorescence visible on FAF imaging (Fig 6). Patient 6 showed the same irregular appearance of the outer segments centrally over a 13-month period, but with fewer apparent dark spaces of nonwaveguiding cones. The inner segment cone mosaic also revealed similar changes at this stage, because the parafoveal cones appeared enlarged compared with those of surrounding areas. Patients 5 and 6 both showed a similar pattern of degeneration over the course of their follow-up that seemed to affect the parafoveal photoreceptor mosaic selectively while sparing the foveal region.

### Electroretinography

**Figure 8:**
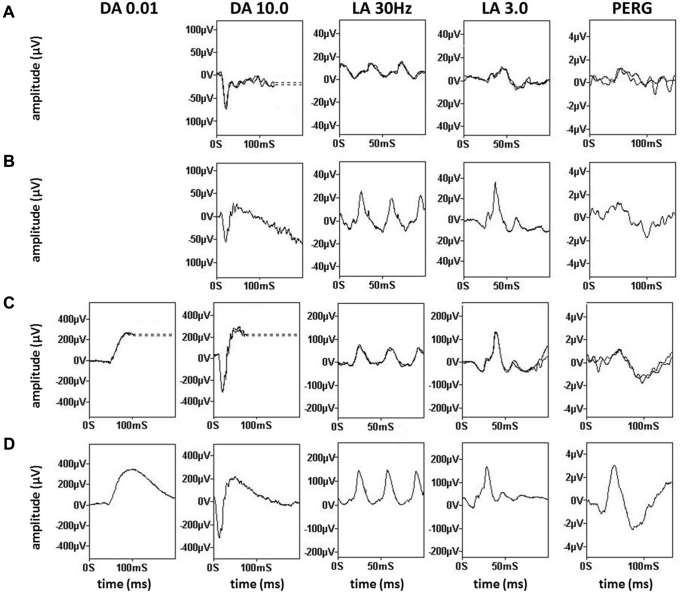

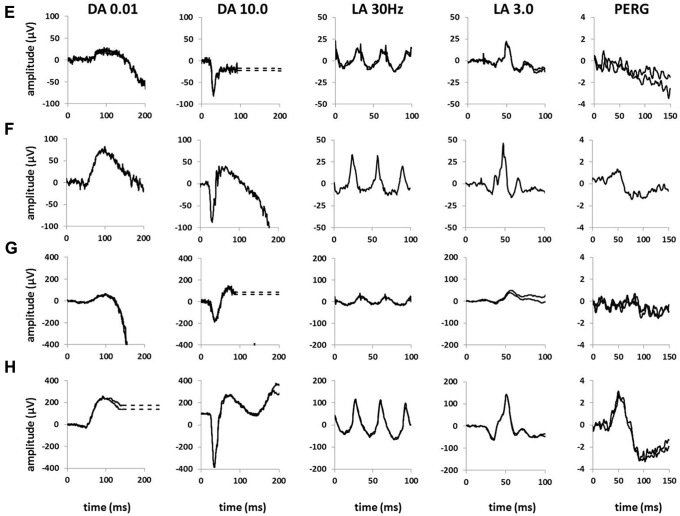
Full-field electroretinography and pattern electroretinography in children with early-stage *ABCA4*-associated retinopathy. Recordings obtained with lower eyelid skin electrodes are shown for (**A**) patient 1 and (**E**) patient 2 and are compared with representative normal traces obtained with the same recording system (**B** and **F**, respectively). Recordings obtained with gold-foil corneal electrodes are shown for (**C**) patient 5 and (**G**) patient 7 and are compared with representative normal traces obtained with the same type of recording system (**D** and **H**, respectively). Dark-adapted (DA) responses are shown for flash strengths of 0.01 and 10.0 cd.s.m^–2^ (DA, 0.01; DA, 10.0) and for light-adapted (LA) electroretinography results for flash strength of 3.0 cd.s.m^−2^ (LA 3.0; 30 Hz and 2 Hz). Pattern electroretinography (PERG) is recorded to an alternating checkerboard. There is a reduced B-to-A ratio (electronegative results) in recordings from (**A**) patient 1 and (**E**) patient 2 consistent with dysfunction that occurs after phototransduction involving the (**A**) rod and cone or (**E**) rod system. Full-field electroretinography results (**C**) are normal for patient 5 and (**G**) show evidence of a cone–rod dystrophy in patient 7. Pattern electroretinography P50 reduction indicates macular dysfunction for patients 2, 5, and 7. All recordings showed a high degree of interocular symmetry and are illustrated for 1 eye only. Note the prestimulus delay in all single flash recordings with the exception of (**H**). For clarity, broken lines replace blink artefacts.

Pattern and full-field electroretinography were performed in 5 of 8 patients (Fig 8). In 3 of 5 patients (patients 2, 5, and 7), the PERG P50 components were subnormal, indicating macular dysfunction. Of the 2 patients with normal PERG results, one was aware of reduced central vision, whereas the other was asymptomatic. Four of 5 patients showed full-field electroretinography evidence of generalized retinal dysfunction despite a normal peripheral retinal appearance on biomicroscopy. Patient 7 showed a reduction in the dark-adapted (DA) strong flash (DA 10.0) electroretinography A-wave and delay and reduction in the light-adapted (LA) 30-Hz flicker electroretinography and LA 3.0 single-flash electroretinography A- and B-waves, consistent with a cone–rod dystrophy. In 2 other patients, DA 10.0 electroretinography results showed normal A-waves, but electronegative waveforms (B-to-A ratio, <1; patients 1 and 2) or a borderline B-to-A ratio (patient 4); there was additionally a low B-to-A ratio in the LA 3.0 electroretinography results in patients 1 and 4 (Fig S1, available at www.aaojournal.org). The electroretinography results in these 3 patients suggest generalized retinal dysfunction occurring after phototransduction involving the rod (patient 2) or both cone and rod systems (patients 1 and 4).

## Discussion

More than 100 years ago, Stargardt^13^ described a series of patients with hereditary visual loss and macular atrophy. Since then, and despite many significant advances, the sequence of events that ultimately result in RPE and photoreceptor cell death remains elusive. In an attempt to address this, we chose to study the earliest stages of STGD1, examining a cohort of children before the development of macular atrophy. Using complimentary structural and functional tests (OCT, FAF, AOSLO, electroretinography), we have been able to identify a number of novel characteristics.

**Figure 9:**
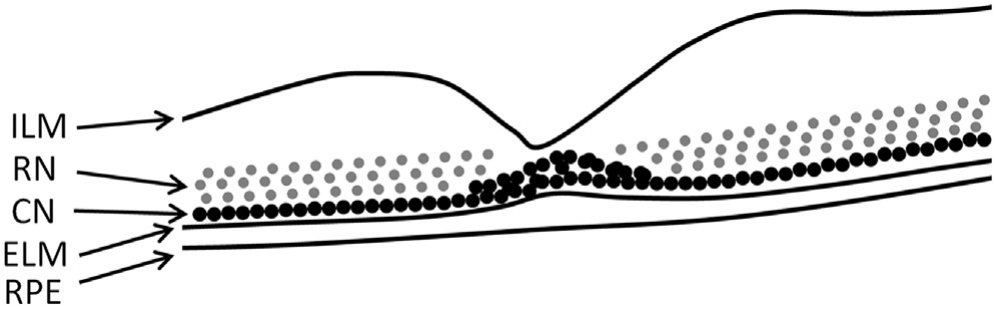
Schematic retinal tomograph highlighting the location of photoreceptor nuclei. Cone nuclei lie within the basal outer nuclear layer (ONL), closely opposed to the external limiting membrane (ELM), except at the foveola, where they are most numerous. Rod nuclei (RN) are less spatially restricted, distributed throughout the entire ONL. The hyperreflectivity visible using OCT seems to correlate directly with the location of cone nuclei (CN). ILM = internal limiting membrane; RPE = retinal pigment epithelium.

One of the earliest structural changes observed was in the ONL, evident on the OCT images of all patients. The OCT images revealed an obvious increase in reflectivity extending internally from the line thought to be the optical correlate of the ELM.^14^ The thickness of this change was at a maximum at the foveola and appeared to decline monotonically as a function of eccentricity to become invisible at the edge of the foveal avascular zone (as defined by the near-infrared reflectance images). Reflectivity within the inner (most vitreal) ONL, a region where Henle fibers reside, always remained unaffected. This uncommon OCT abnormality has been termed *ELM thickening*, presumably because of the increased reflectivity appearing to be continuous with the ELM.^11^, ^15^, ^16^, ^17^, ^18^ The ELM represents the adherens junctions between Müller cells, or Müller cells and photoreceptor inner segments, and it seems improbable that thickening of this structure would occur as the first sign of STGD1.^19^ We now suggest an alternative hypothesis: that the identified changes are the result of pathologic disruption within the outer lamella of the ONL, specifically in the cones. Within the ONL, cone photoreceptor nuclei have defined spatial distribution, being most numerous and tightly packed at the fovea and less densely packed, residing more closely approximated to the ELM, in the perifoveal retina (Fig 9).^20^ In contrast, rod nuclei are thought to be less spatially restricted and are distributed throughout the entire thickness of the ONL.^20^ This arrangement of cone and rod nuclei has been known for more than a century and originally was described by Ramón y Cajal and Swanson.^21^ More recent studies have shown the same distribution in murine and primate retinas.^22^, ^23^ OCT imaging in this case series confirmed that the ellipsoid zone is still evident in early disease, but it appears to be reduced qualitatively in intensity, suggesting that in addition to the changes to the ONL, there are subtle outer segment pathologic features. Finally, increased ONL reflectivity seems to be a transient phenomenon, sustained only as long as the ONL volume is preserved, whereas the line representing the ELM remains preserved at the same stage (Fig 6). Indeed, others have commented on the robust nature of the ELM, even in patients with advanced disease.^15^

Sequential imaging provided further insight into the precise sequence of events leading to macular atrophy. Where significant numbers of photoreceptor nuclei were lost, OCT-documented collapse of the inner retinal layers into ONL seemed to occur preferentially at perifoveal loci, sparing the foveola, at least initially (Figs 4 and 5). This pattern of degeneration also was evident using the split-detector AOSLO, where the greatest increase in cone spacing occurred in the parafoveal region, with relative sparing of the foveolar cones (Figs 5 and 6). However, quantification of the AOSLO changes was not possible, both because of the difficult nature of imaging young patients and the size and dense packing of foveal cones at baseline visits, which were comparable with those of unaffected individuals. OCT determined ONL thickness measurements therefore may be the current biomarker of choice for monitoring this group of patients. Regardless of the imaging technique used, foveolar-sparing retinal degeneration was a particularly unexpected finding, because foveal involvement usually is the hallmark of childhood-onset STGD1. However, if individuals are imaged with sufficient resolution at an early stage of disease, foveola sparing may be the rule rather than the exception. Because childhood-onset disease usually evolves rapidly, the best chance of identifying this stage of disease is likely to be provided by examining siblings of affected children, or less commonly as observed here, when peripheral flecks are detected on a routine eye examination at a time when visual acuity is preserved temporarily. Early parafoveal involvement also is a notable feature of another STGD1 phenotype, a late-onset adult form of STGD1 in which foveal sparing is common.^24^ Although childhood-onset disease with early foveal involvement and adult-onset foveal-sparing disease are likely to represent 2 extremes of the STGD1 severity spectrum, both seem to share a similar pattern of retinal degeneration and differ only in the rate at which their disease progresses. Finally, if foveolar-sparing disease is the norm, one could counter this assertion arguing that a specific subgroup of patients exist where degeneration begins at the fovea: those with outer retinal cavitation.^25^ High-resolution imaging in patient 5 demonstrated that even when photoreceptors are not visible using OCT, they can be detected using AOSLO, and that even at this time, residual cones are still most numerous at the foveola (Fig 6). Our findings therefore suggest that therapeutic interventions are likely to work best in childhood-onset *ABCA4*-associated retinopathy if given very early, although it may be challenging to recruit such patients for clinical trials until convincing safety and efficacy data are available in adults.

Autofluorescence imaging additionally contributes novel information. In keeping with the hypothesis that perifoveal disease occurs first, foveolar autofluorescence characteristics were relatively well preserved, because all individuals retained a degree of physiological hypoautofluorescence. This suggests that structures important for imparting these characteristics, the RPE and macular pigments within Henle fibre layer, also remain relatively intact.^26^ Fundus autofluorescence imaging also was able to characterize 2 discrete patterns of hyperautofluorescence: classical pisciform flecks that do not encroach on the foveola and tiny discrete dots that never extend outside the foveola (Fig 2). Although both lesions eventually evolve to outer retinal atrophy, they may do so at different rates. Whereas the larger outer retinal deposits are visible using OCT, the precise location of the smaller foveal lesions remains unknown. Although the present work cannot answer this question definitively, it is tempting to speculate that these characteristics may result from differences in photoreceptor retinal metabolism, via an intraretinal pathway involving Müller cells for cones or via the RPE for rods.^27^ If true, this would mean that the tiny hyperautofluorescent dots are likely to be intraretinal, whereas the larger flecks are present in the RPE–photoreceptor outer segments. This is in keeping with prior studies that have identified autofluorescent material in cone inner segments and in Müller cell processes, as well as more conventionally in the RPE.^28^, ^29^ Our OCT and FAF data also support the hypothesis that cone photoreceptor dysfunction is intrinsic and not secondary to RPE failure, concurring with previous animal studies.^30^ In the absence of *ABCA4* flippase activity, toxic bisretinoids accumulate in the photoreceptor outer segment, which occurs to a greater extent in mice with a cone-dominant (Abca4^−/−^/Nrl^−/−^) compared with a rod-dominant (Abca4^−/−^) background.^30^ Not only is bisretinoid production higher in cones than in rods, cones are also slower to clear this, transferring less of this toxic product to the RPE.^30^ Therefore, it is unsurprising that these biochemical differences are reflected in the clinical signs.

The electroretinography findings in 4 of 5 children indicated generalized retinal dysfunction, including 3 patients with electronegative findings, in keeping with a locus of dysfunction occurring after phototransduction. Subnormal electroretinography B-to-A ratios and electronegative findings (B-to-A ratio less than 1.0) have been reported in a wide range of inherited retinal diseases, but not consistently in STGD1.^31^ A rare published example was in an 8-year-old boy with early-onset panretinal STGD1.^32^ Because all children in the current study had mild or early disease, it is postulated that the electroretinography findings may arise from metabolically stressed photoreceptors, before cell death, with the most sensitive physiologic processes being the most vulnerable. Phototransduction and outer segment hyperpolarization may remain relatively well preserved, consistent with electroretinography A-wave preservation, whereas inner segment physiology, in particular metabolically demanding processes such as neurotransmitter release from the presynaptic terminal, may be compromised. Disruption of neurotransmitter release would impair signaling to both the rod-on and cone-on and -off bipolar cells, largely responsible for generation of DA and LA electroretinography B-waves, respectively. The same mechanism also may contribute to the electroretinography electronegativity observed in some other primary photoreceptor dystrophies.^33^ Alternative explanations for a low B-to-A ratio include retraction of photoreceptor synapses and de novo synapse formation, with other cells in the inner retina leading to disrupted signaling to the bipolar cells.

Finally, 2 disease-associated alleles were identified in all patients in this series, highlighting the prior observation that genetic testing in childhood-onset STGD1 has a high chance of success.^17^, ^34^ Again in keeping with previous reports is the identification of the allele c.5461–10T→C, which recurred here in 2 of 8 patients. This variant now has been identified consistently in childhood-onset cohorts, in trans with missense variants, as well as in association with late-onset foveal-sparing disease, where the second allele often remains undetected.^2^, ^17^, ^34^, ^35^ The most recent evidence suggests that this variant is itself pathogenic and not necessarily in linkage disequilibrium with an as-yet undiscovered disease-causing allele.^36^, ^37^ To the best of our knowledge, the same combination of *ABCA4* alleles has not been reported to cause mild (late-onset, foveal-sparing) and severe disease.

This work represents the first detailed description of the earliest anatomic changes that occur in childhood-onset *ABCA4*-associated retinopathy. Rather than using an arbitrary cutoff based on patient age, we defined the earliest stages by the absence of clinically apparent outer retinal atrophy. As a result, most individuals were minimally symptomatic. Using this approach, we have been able to highlight novel features associated with early photoreceptor degeneration. Why the foveola is relatively spared in the initial stages of disease is yet to be determined, although one may speculate that this relates to structural or functional differences between foveal and parafoveal cones. These findings will facilitate detection of STGD1 in its mildest form and will provide vision researchers with novel insights into the first stages of disease. Our hope is that this work also advances the identification and verification of sensitive biomarkers necessary for monitoring patient outcomes in future therapeutic trials.
