# Supplementary material for: Early Patterns of Macular Degeneration in ABCA4-Associated Retinopathy
Source: Ophthalmology. 2018 May;125(5):735–46. doi: 10.1016/j.ophtha.2017.11.020 (PMC5917070; doi:10.1016/j.ophtha.2017.11.020)
Supplement: Figure S1 [file mmc1.pdf]

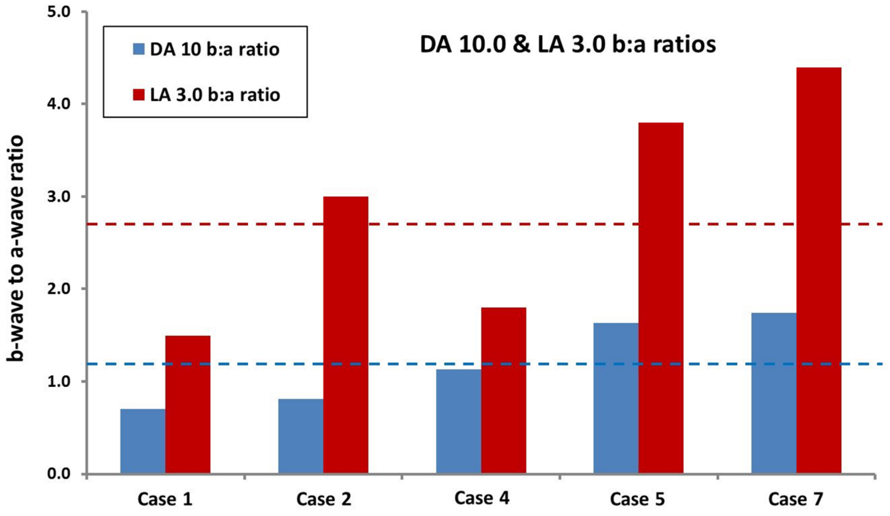

Supplementary Figure 1.
Summary of the DA10.0 ERG and LA 3.0 ERG b:a amplitude ratios in five children with *ABCA4*-associated retinopathy. Broken lines show 5th percentile for DA 10.0 ERG (blue line) and LA 3.0 ERG b:a ratios (red line). There is a reduced or low b:a ratio in 3 of 5 cases, consistent with dysfunction occurring after phototransduction.
