# Supplementary material for: Early Patterns of Macular Degeneration in ABCA4-Associated Retinopathy
Source: Ophthalmology. 2018 May;125(5):735–46. doi: 10.1016/j.ophtha.2017.11.020 (PMC5917070; doi:10.1016/j.ophtha.2017.11.020)
Supplement: Table S1 [file mmc2.pdf]

ID	Age at exam (years)	VA [RE, LE] (logMAR)	Fundoscopy	FAF	OCT	Electrophysiology	Genetics	Variant frequency in other childhood STGD1 cohorts
12149124	5<br><br>6	0.3, 0.26<br><br>0.22, 0.22	Abnormal foveal reflex	Perifoveal rhomboid of increased AF.	Hyper-reflectivity at base of ONL<br><br>Evolution to perifoveal atrophy 12 months later	Skin electrodes<br><br>PERG P50 normal.<br><br>DA 10 ERG a-wave normal; b:a ratio subnormal (electronegative)<br>LA 30Hz ERG delayed<br>LA 3.0 ERG delayed; b:a ratio reduced	c.214G>A, p.Gly72Arg;<br>c.214G>A, p.Gly72Arg	1% [10]<br>1% [10]
21947341	5<br><br>6	0.24, 0.26 (0.1, 0.1 non-illuminated Kay picture test)<br><br>0.4, 0.3	Abnormal foveal reflex	Subtle increase in perifoveolar AF, center retains hypoAF.	Hyper-reflectivity at base of ONL, loss of EZ juxtafoveal<br><br>Loss of perifoveal outer retinal volume 12 months later	Skin electrodes<br><br>PERG P50 undetectable<br><br>DA 0.01 ERG subnormal<br>DA 10.0 ERG ERG a-wave normal; b:a ratio subnormal (electronegative)<br><br>LA 30Hz & LA 3.0 ERG normal	c.4469G>A, p.Cys1490Tyr;<br>c.5461-10T>C	6% [11]<br>22% [10]
31625765	9<br><br>11	0.07, 0.07<br><br>0.98, 1.0	Widespread peripheral flecks and altered foveal reflex	Retained central hypoAF zone but reduced in size, dots of increased AF (central foveola is spared)	Hyper-reflectivity at base of ONL, loss of EZ and temporal “collapse”	Not performed	c.768G>T, p.Val256Val;<br>c.4363T>C, p.Cys1455Arg	13% [10]<br>3% [11]

42215152	13<br><br><br>14	0, 0.1<br><br><br>0.36, 0.36	Normal	Mild increase in perifoveolar AF with more diffuse peripheral macular increase in AF	Hyper-reflectivity at base of ONL, loss of EZ juxtafoveal<br><br><br>Loss of perifoveal outer retinal volume 12 months later, temporal > nasal	Corneal electrodes<br><br>PERG P50 normal.<br>DA 0.01 ERG normal<br>DA 10.0 ERG a-wave normal;<br>b:a ratio borderline<br><br>LA 30Hz ERG timing borderline; amplitude normal<br>LA 3.0 ERG b:a ratio subnormal	c. 6320G>C, p.Arg2107Cys;<br>c.5461-10T>C	2% [10]<br>22% [10]
51927327	8<br><br><br>11	0.2, 0.2<br><br><br>0.24, 0.3	Abnormal foveal reflex	Tiny zone of central hypoAF surrounded by broader zone of increased AF, a few central dots of increased AF	More subtle hyper-reflectivity at base of ONL extending into the perifoveal region, widespread ONL thinning including at foveola, perifoveal "collapse". Later evolution to flat cavitation.	Corneal electrodes<br><br>PERG P50 subnormal.<br><br>DA 0.01 & DA10.0 ERG normal<br><br>LA 30Hz & LA 3.0 ERG normal	c.2588G>C, p.Gly863Ala;<br>c.5161_5162delAC	4% [10]<br>1% [10]
61879914	11	-0.140, -0.120	Subtle yellow-white central macular dots	Retained central hypoAF zone but reduced in size, dots of increased AF surrounded by a zone of more diffuse increased AF	Clearly demarcated hyper-reflectivity at the base of the ONL sparing Henle nerve fibre layer, loss of ONL thickness, loss of integrity of EZ.	Not performed	c.2521C>T, p.Gln841Ter;<br>c.6097C>T, p.Leu2027Phe	Not reported<br>4% [11]

71635876 (previously reported in 10.1111/aos.12259)	7<br><br>8<br><br>10<br><br>12	0.3, 0.375 (2011)<br>0.4, 0.4 (2012)<br>1.0, 0.9 (2014)<br>1.0, 0.9 (2016)	Normal initially, followed by the development of yellow foveal dots.	Subtle rhomboid-shaped diffuse increased zone of autofluorescence. Hyperautofluorescent dots are absent from the fovea, but present in the perifovea, corresponding to regions out POS atrophy.		Corneal electrodes<br><br>PERG 50 subnormal<br><br>DA0.01 ERG subnormal<br>DA10.0 ERG a-wave subnormal (b:a ratio normal)<br><br>LA 30Hz & LA 3.0 ERG delayed and subnormal (b:a ratio normal)	c.6729+5_6729+19del15 hom	Identified in a young adult with very severe disease aged 30. [36]
8K1	9<br><br>12	0.1, 0.15<br><br>0.5, 0.6	Normal<br><br>Yellow flecks outside the vascular arcades	Retained central hypoAF zone but reduced in size, dots of increased AF in a rhomboid-shaped zone of more diffuse increased AF<br><br>Tiny central island of low AF, surrounded by irregular areas of increased AF	Clearly demarcated hyper-reflectivity at the base of the ONL sparing Henle nerve fibre layer, maximal at the foveola, reducing in the the peri-fovea.<br><br>Loss of volume most evident in perifoveal outer retinal structures (sclerad to the ELM). Foveal photoreceptor outer segments appear to be the last to degenerate.	Not performed	c.3056C>T, p.Thr1019Met;<br>c.4463G>A, p.Cys1488Arg	2% [10]<br>1% [11]

RE = right eye, LE = left eye, AF = autofluorescence, FAF = fundus autofluorescence, OCT = optical coherence tomography, ONL = outer nuclear layer, EZ = ellipsoid zone, PERG = pattern electroretinogram, ERG = full-field electroretinogram, DA10.0 = dark-adapted bright flash electroretinogram, [10] = reference 10, [11] = reference 11.
